# A Lipidomics Approach in the Characterization of Zika-Infected Mosquito Cells: Potential Targets for Breaking the Transmission Cycle

**DOI:** 10.1371/journal.pone.0164377

**Published:** 2016-10-10

**Authors:** Carlos Fernando Odir Rodrigues Melo, Diogo Noin de Oliveira, Estela de Oliveira Lima, Tatiane Melina Guerreiro, Cibele Zanardi Esteves, Raissa Marques Beck, Marina Aiello Padilla, Guilherme Paier Milanez, Clarice Weis Arns, José Luiz Proença-Modena, Jayme Augusto Souza-Neto, Rodrigo Ramos Catharino

**Affiliations:** 1 Innovare Biomarkers Laboratory, School of Pharmaceutical Sciences, University of Campinas, Campinas, 13083-877, Brazil; 2 Animal viruses Laboratory, Department of Genetics, Evolution and Bioagents, Institute of Biology, University of Campinas, Campinas, 13083-862, Brazil; 3 Emerging viruses study Laboratory, Department of Genetics, Evolution and Bioagents, Institute of Biology, University of Campinas, Campinas, 13083-862, Brazil; 4 Vector Functional Genomics & Microbiology Laboratory, UNESP Institute of Biotechnology, São Paulo State University, Alameda das Tecomarias s/n, Botucatu, 18607-440, Brazil; 5 Department of Bioprocesses and Biotechnology, Faculty of Agronomical Sciences, São Paulo State University, Rua José Barbosa 1780, Botucatu, 18610-307, Brazil; Instituto Nacional de Salud Pública, MEXICO

## Abstract

Recent outbreaks of Zika virus in Oceania and Latin America, accompanied by unexpected clinical complications, made this infection a global public health concern. This virus has tropism to neural tissue, leading to microcephaly in newborns in a significant proportion of infected mothers. The clinical relevance of this infection, the difficulty to perform accurate diagnosis and the small amount of data in literature indicate the necessity of studies on Zika infection in order to characterize new biomarkers of this infection and to establish new targets for viral control in vertebrates and invertebrate vectors. Thus, this study aims at establishing a lipidomics profile of infected mosquito cells compared to a control group to define potential targets for viral control in mosquitoes. Thirteen lipids were elected as specific markers for Zika virus infection (Brazilian strain), which were identified as putatively linked to the intracellular mechanism of viral replication and/or cell recognition. Our findings bring biochemical information that may translate into useful targets for breaking the transmission cycle.

## Introduction

Zika virus (ZIKV) is an emerging arbovirus that is transmitted by mosquitoes of the genus *Aedes* [1], and was first isolated in 1947 in eastern Africa, remaining restricted to the African and Asian continents until 2007, where it was seldom observed in humans [2]. Typically, the infection of ZIKV in humans is either asymptomatic or associated with a self-limiting febrile illness, in only 20% of infected people. However, recent outbreaks of ZIKV in South Pacific and Latin America have evidenced the virus potential to cause severe neurological damage-associated complications such as Guillain-Barré syndrome [3] and microcephaly in newborns [4].

Similarly to dengue virus (DENV), ZIKV is an enveloped, single-stranded, positive RNA virus whose 10.7-kb genome encodes three structural proteins (C, capsid; M, membrane; and E, envelope) and seven nonstructural proteins (NS1, NS2a, NS2b, NS3, NS4a, NS4b and NS5) [2, 5]. E protein is a major ZIKV antigen, which coordinates the association between the virion and the host’s viral receptors and membrane lipids [6, 7]. Recent studies have demonstrated that viral and host lipids play an important role in the attachment process during viral infection. These lipids, adhered to the capsid surface, coordinate viral recognition allowing its entry into the host cell [8–13].

Intracellular host cell membranes multiply and reorganize during infection in order to form viral replication complexes (VRCs), leading to the accumulation of phosphatidylcholine (PC) in VRCs, as demonstrated for Poliovirus and Hepatitis C [14]. Furthermore, VRC biogenesis requires increased membrane fluidity in order to facilitate viral RNA transfer throughout pores formed on the packing vesicles [14]. Strikingly, a recent lipidomics study has demonstrated that intracellular membrane alterations induced by DENV are intimately associated with a set of lipids uniquely found on DENV-infected mosquito cells, especially in association with VCR membranes [15], highlighting the crucial role of such molecules in this process.

On the other hand, lipid droplets (LDs) have been recently pointed out as an important component of the *A*. *aegypti* antiviral defenses [16], which also rely on the RNAi machinery [17] and innate immune pathways Toll and JAK-STAT [18, 19] to contain viral replication. LDs accumulate upon bacterial and viral infections in both adult mosquito midgut and cell lines, in a process that seems to be associated with NF-k-B immune pathways activation with participation of the insect gut microbiota [16].

While much progress has been achieved in the past decade towards understanding the mosquito’s transcriptional and metabolic responses to DENV infection, mosquito-ZIKV interactions continue largely unknown. In addition to the limitations of both clinical and laboratory diagnosis and the absence of a specific treatment for ZIKV infection [2, 20, 21], this poses a major challenge for the development of control interventions.

The present study aims at verifying the alterations in the mosquito cell lipidome during ZIKV infection using the MALDI Mass Spectrometry Imaging (MALDI-MSI) approach in order to identify and characterize important molecules associated with *Aedes*-ZIKV interactions. Our findings indicate that, as with DENV infection [15], *Aedes’* cells increase their glycerophospholipid metabolism for some lipids, which may represent potential targets for blocking viral replication in mosquitoes or for further developments in novel therapeutic approaches in humans, since it is known that some factors required for viral infection are conserved among *Diptera* and human hosts [22].

## Methods

### Cell culture

The *Aedes albopictus* C6/36 cell line (ATCC^®^ CRL-1660) was cultured in special Leibovitz L-15 medium (Vitrocell^®^), with 1% of essential amino acids, pyruvate, penicillin, streptomycin, and amphotericin (SigmalAldrich) and 10% of bovine fetal serum—BFS (Vitrocell^®^). These cells were conditioned at 28°C with 5% of CO_2_ at the Animal Virology Laboratory of the University of Campinas.

### Zika virus isolate

Brazilian ZIVK strain (BeH823339, GenBank KU729217) was kindly provided by Professor Edison Durigon (Biomedical Sciences Institute, University of São Paulo). This viral strain was originally isolated by a team of the Evandro Chagas Institute, Pará State, Brazil from a Ceará’s State (Brazil) patient in 2015. The virus was stored at -80°C in the Animal Virology Laboratory of the University of Campinas until use. Before procedure, the viral titer in this stock was determined by PFU assay in Vero cells.

### Infection of A. albopictus C6/36 cells with zika virus

For ZIKV infection, 1x10^5^ cells of C6/36 cell line was added to each well of a 24-well plate, and incubated for 24 hours at 28°C with 5% of CO_2_. Viral Infection was performed using a multiplicity of infection (MOI) of 10. The plate was then incubated under 5% of CO_2_ at room temperature for five days. The choice of this timepoint was given because this was when cytopathic effects began to arise in the cell culture [23, 24]. The same procedure was carried out for the negative control samples, without ZIKV inoculation, resulting in 15 biological replicates for each condition studied.

### Viral detection by RT-qPCR

In order to confirm ZIKV infection, the viral stock and the supernatant of ZIKV-infected C6/36 cells were assayed by real time RT-qPCR [25]. Briefly, the viral RNA was isolated by a commercial kit following the manufacturer’s instruction (RNeasy Mini Kit, Qiagen, Hilden, Germany). One step RT-PCR amplification of viral RNA (Taqman RNA to-CT, Applied Biosystems) was performed with following primers and probes: ZIKV-F: 5’- CCGCTGCCCAACACAAG-3’; ZIKV-R: 5’- CCACTAACGTTCTTTTGCAGACAT -3’; ZIKV-P: 5’-/FAM/AGCCTACCTTGACAAGCAGTCAGACACTCAA/-3’. All reactions were assembled in a final volume of 12.5 μL with 300 ng of RNA, 1 PrimeTime mix (Integrated DNA Technologies) containing both primers and probe, and 6.25 μL of TaqMan master mix (Applied Biosystems) by using the following cycling algorithm: 48°C for 30 min, 95°C for 10 min, followed by 45 cycles of 95°C for 15 s and 60°C for 1 min.

### MALDI-MSI analysis

Six days after infection, cells were placed on glass slides 24x60mm and covered with MALDI matrix α-cyano-4-hydroxycinnamic acid (Sigma-Aldrich, St. Louis, MO) solution at 10 mg/mL in 1:1 acetonitrile/methanol. Spectra were acquired using a MALDI LTQ-XL (Thermo Scientific, San Jose, CA) at the mass range of 400 to 1200 *m/z*, in the negative ion mode for a total of 15 replicates per group. MS/MS data were acquired in the same instrument, using Helium as the collision gas, with energies for collision-induced dissociation (CID) ranging from 20–80 (arbitrary units). Spectra were analyzed using XCalibur software (v. 2.4, Thermo Scientific, San Jose, CA).

### Structural elucidation

Lipids were identified based on the analysis of the MS/MS fragmentation profile of the ions selected by statistical analyses. Structures were proposed using theoretical calculations modeling for molecular fragmentation using Mass Frontier software (v. 6.0, Thermo Scientific, San Jose, CA).

### Semi quantification by MSI

Chemical images of the markers were generated using the imaging feature of MALDI and processed to grayscale using the ImageQuest software (Thermo Scientific, San Jose, CA). All samples, control and ZIKV, and each respective replicate (n = 15) had their chemical image standardized and analyzed using ImageJ software (National Institutes of Health, USA—open source). A non-dimensional value was assigned to each image based on pixel intensity, so that the intensity/quantity ratio is established [26].

### Statistical analysis

Partial least squares discriminant analysis (PLS-DA) was used as the method of choice to assess association between groups; this supervised method uses multivariate regression techniques to extract, through linear combination of the original variables, the characteristics that may evidence this association. Statistical significance of the obtained model was assessed by using two permutation tests: precision preview during modeling and separation distance; 2000 permutations were used in both tests. The selection of lipids that were characteristic for each sample was carried out considering the impact that each metabolite had in the analysis through VIP (Variable Importance in Projection) scores, which consists of the weighted average of the squares of the PLS loadings, and takes into account the amount of explained variance on each dimension used in the model. As a cutoff threshold, only the chemical markers with a VIP score greater than 1.5 were analyzed. The heat map of the VIPs was built using the Pearson’s distance measurement and Ward’s clustering algorithm. All analyses involving PLS-DA and VIP scores were carried out using the online software MetaboAnalyst 3.0 [27].

For comparative purposes of semi-quantitative data, either Student’s t-test or Mann-Whitney’s U-test was used after applying Kolmogorov-Smirnov normal distribution test; a p-value was significant if it presented values lower than 0.05. All those calculations were carried out using the software GraphPad Prism (v.3.0, GraphPad Software, San Diego, CA).

## Results

**Fig 1:**
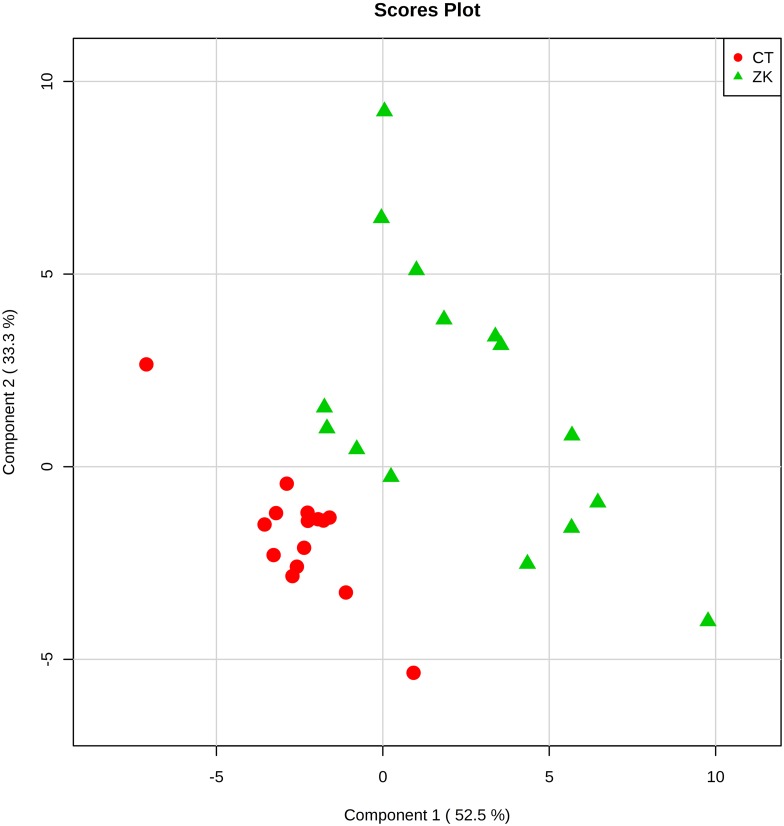
Scores plot between the first two principal components (PCs) selected. The explained variances are 33.5% for component 1 and 52.5% for component two.

**Fig 2:**
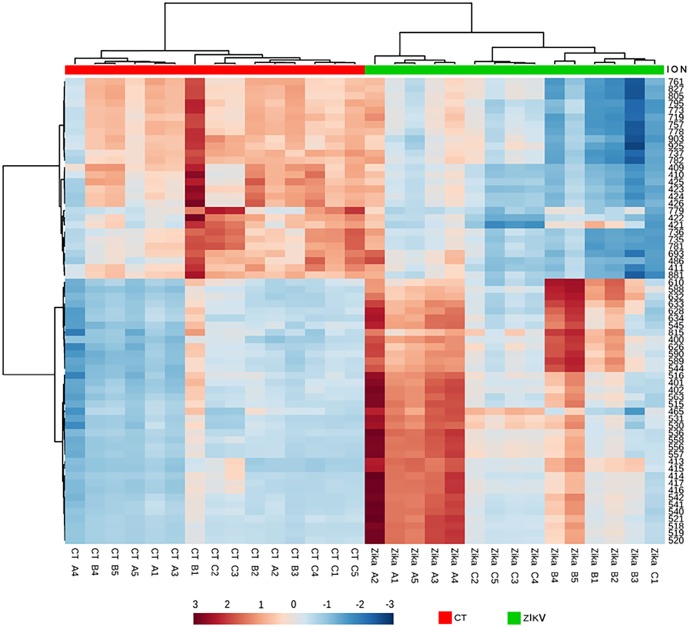
Clustering result for the 65 top features in the PLS-DA VIP scores, shown as a heat map (distance measured by Euclidean and clustering algorithm using ward.D), with a color-coded thermometer (bottom) indicating the relative concentrations of metabolites on each respective group.

In order to analyze the main metabolites involved with ZIKV infection in the mosquito, we performed a metabolomic analysis on *Aedes albopictus* C6/36 cells, either infected by the Brazilian ZIKV strain, or uninfected. Our data suggest that a large amount of lipids is involved in the responses to ZIKV infection. A PLS-DA statistical analysis of our mass spectrometry-derived data evidenced a clear separation between the metabolite composition in ZIKV-infected versus uninfected C6/36 mosquito control cells, as demonstrated in Fig 1; statistical validation of this PLS-DA model by permutation tests are provided on S1 Fig (prediction accuracy) and S2 Fig (prediction accuracy during training). Using VIP scores, 65 features were identified with a score value greater or equal to 1.5, as demonstrated in Fig 2; the ZIKV-infected cells accounted for 37 characteristic markers whereas the uninfected control ones displayed 28 molecules. Out of these 65 features, 20 lipid markers were identify using *tandem* mass spectrometry (MS/MS), divided in 13 species for the ZIKV-infected group (Table 1) and 7 for the control group (Table 2).

**Table 1:** Lipid markers elected by PLS-DA VIP scores ≥ 1.5 and elucidated by MS/MS for *Aedes albopictus* C6/36 infected cells (ZIKV group).

Class	Ion (*m/z*)	Molecule	MS/MS
Sphingolipid	400	Sphingofungin F	311, 336, 356
Phosphatidylserine	516	PS(18:4(6Z,9Z,12Z,15Z)/0:0)	272, 289, 326, 345
	518	PS(18:3(9Z,12Z,15Z)/0:0)	373, 387, 429, 474
	520	PS(18:2(9Z,12Z)/0:0)	375, 429, 431, 476
	540	PS(20:6(2Z,5Z,8Z,11Z,14Z,17Z)/0:0)	373, 395, 400, 491, 496, 522
	542	PS(20:5(5Z,8Z,11Z,14Z,17Z)/0:0)	375, 402, 493, 498
	544	PS(20:4(5Z,8Z,11Z,14Z)/0:0)	355, 399, 456, 500
Phosphatidylcholine	530	PC(19:3(10Z,13Z,16Z)/0:0)	472, 512
	536	PC(19:0/0:0)	311, 404, 445, 447
	556	PC(6:2(3E,5E)/14:2(11E,13E))	373, 389, 400, 416, 512
	558	PC(6:2(3E,5E)/14:1(13E))	375, 402, 412, 418, 514
Dyacylglicerole	563	DG(16:1(9Z)/16:1(9Z)/0:0)	519, 537, 545
Phosphatidylethanolamine	632	PE(12:0/16:1(9Z))	544, 562, 588

**Table 2:** Lipid markers elect by PLD-DA VIP score ≥ 1.5 and elucidated by MS/MS for *Aedes albopictus* C6/36 uninfected cells (Control group).

Class	Ion (m/z)	Molecule	MS/MS
Phosphatidic Acid	409	PA(16:0/0:0)	263, 365, 391
Phosphoethanolamine	757	PE(18:2(9Z,12Z)/19:0)	713, 730, 739
	773	PE(20:0/18:1(9Z))	627, 645, 728, 755
	779	PE(16:0/22:6(54Z,7Z,10Z,12E,16Z,19Z)(14OH))	502, 546, 589, 735
Phosphatidylserine	881	PS(22:4(7Z,10Z,13Z,16Z)/21:0)	736, 836, 863,
	903	PS(22:0/22:0)	714, 758, 858, 884
Triacylglycerol	925	TG(18:2(9Z,12Z)/20:4(5Z,8Z,11Z,14Z)/20:4(5Z,8Z,11Z,14Z))	881, 899, 907

Of the 13 lipids species indentified for ZIKV-infected cells (Table 1), 12 participate directly in the intracellular metabolism of glycerophospholipid in the host cells: 8 are acylglycerophospholipids, divided into 6 acylglycerophosphoserines and 2 acylglycerophosphocholines; 3 species are diacylglycerophospholipids, divided in two diacylglycerophosphocholines and 1 diacylglycerophosphoserine; 1 is a diacylglycerol; and one is a sphingolipid. Hence, our data suggest that the glycerophospholipid metabolism pathway activity is much increased in the ZIKV-infection group, similarly to other viral infections [14, 15, 28].

**Fig 3:**
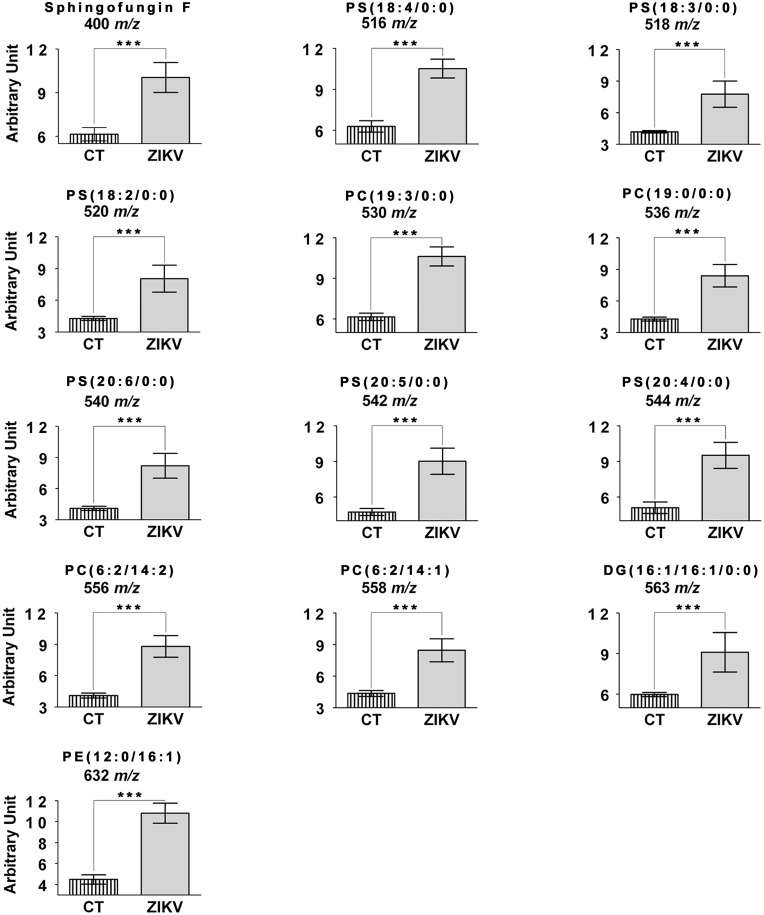
Semi-quantitative analysis of characteristic lipids showed that ZIKV-infected cells. The bars representing confidence interval of 95% (**** p<0.0001)

To corroborate the abovementioned evidence of ZIKV infection interference in the mosquito cells’ metabolism, a semi-quantitative analysis of characteristic lipids confirmed that ZIKV-infected cells presented statistically significant higher levels (p<0.05) of all PLS-DA-elected lipids previously elected as characteristic markers for ZIKV-infection based on the quantitative comparison performed with each marker between ZIKV-infection and control samples (Fig 3). We observed up to 2.15-fold higher concentration of metabolites such as ion at *m/z* 556 [PC(6:2(3E,5E)/14:2(11E,13E))] in ZIKV-infected cells when compared to non infected controls.

**Fig 4:**
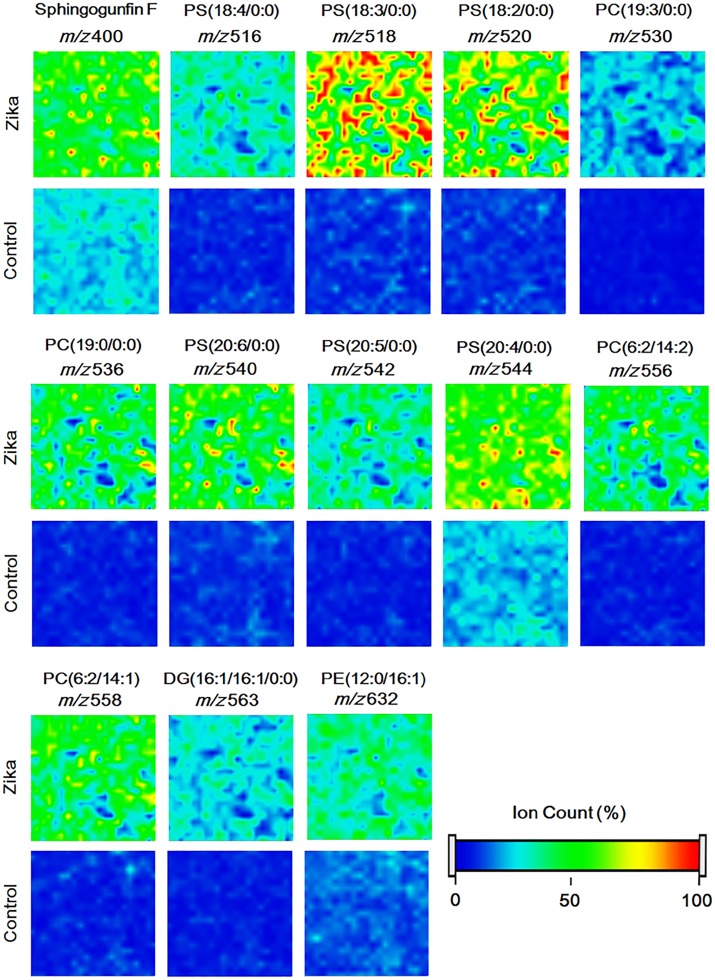
Chemical images from MALDI-MSI showing the comparison between infected and uninfected cells. It is visually possible to assess the difference in lipid composition of ZIKV-specific characterized biomarkers.

Nevertheless, the quantitative comparison between the elected markers for control and ZIKV-infected cells was not statistically significant, despite a slight increase in the amount of elected markers in the control cells was observed (p<0.05, S3 Fig), suggesting that the same glycerophospholipid metabolic pathway is active in both the control and the ZIKV-infected cells. This may be explained by the fact that both ZIKV-infected and uninfected cells have essentially the same metabolic profile, except for some specific metabolic changes induced by the virus in the cell line [14, 29]; the regular glycerophospholipid metabolism, however, remain at lower levels when the cell is in homeostasis [30]. Fig 4 presents a comparison between ZIKV-infected and non-infected cells, where it is visually possible to assess the differences in lipid composition of ZIKV-specific characterized biomarkers. The present study uses mosquito cells; however, the host factors identified may be the same as for the human host, since this has been shown in other studies on viral infections [22].

## Discussion

### Sphingolipids (SL)

Sphingofungin F, the ion at *m/z* 400 (Table 1), was the SL found as marker for ZIKV infection. This compound was isolated for the first time during the fermentation of *Paecilomyces variotii* (ATCC 74097), and it is a bioactive lipid that acts as the inhibitor of serine palmitoyltransferase (SPT), an essential enzyme during the biosynthesis of SL (26). The SPT inhibitor was initially investigated for its antifungal activity; however, later studies showed that these inhibitors suppress viral replication, as described with for myriocin-based SPT inhibition in hepatitis C virus (HCV) [29, 31], as well as for 4-hydroxyphenyl retinamide inhibitor in a study with DENV [32]. Like ZIKV, these two viruses also belong to the *Flaviviridae* family. No previous report has ever implicated this SL as an insect metabolite; it has only been associated to fungi metabolism [1]. As observed in Fig 4, this molecule is present in both cultures, but the relative amount in the ZIKV-infected group is 1.64-fold (p<0.0001) higher than in the control group. This may be an indicative that the infected cells are responding to viral infection, in an attempt to impair viral replication.

### Phosphatidylserines (PS)

PS is the most abundant anionic lipid class in the plasma membrane of eukaryotes. It is essentially produced in the inner side of the plasma membrane when the cell is in homeostasis; however, this asymmetry is lost during injury, malignancy, or the apoptosis [33, 34]. Under apoptotic conditions, cells reverse PS to the outer side of the membrane. PS signalize to phagocytes that cell is under apoptosis [35]. The exact processes, by which the cell externalizes these lipids, or how this process is initiated, are not fully understood [36]. Cell cultures were analyzed 7 days after seeding, which marks the beginning of the culture decay process. Hence, the two PS lipids elected as markers for the control group, *m/z* 881 [PS(22:4(7Z,10Z,13Z,16Z)/21:0)] and *m/z* 903 [PS(22:0/22:0)] may be due to the apoptotic process that may have been initiated in the cell culture, which lead to a higher dead cells ratio, as cell cultures are self-limiting in growth. Additionally, since both cultures were analyzed at the same time point, the ZIKV-infected group was likely in the same phase of the control one, which may explain the lack of statistical significance in the quantitative analysis of markers between these two groups.

The level of all 6 PS characterized for the ZIKV-infected culture, *m/z* 516 [PS(18:4(6Z,9Z,12Z,15Z)/0:0)], *m/z* 518 [PS(18:3(9Z,12Z,15Z)/0:0)], *m/z* 520 [PS(18:2(9Z,12Z)/0:0)], *m/z* 540 [PS(20:6(2Z,5Z,8Z,11Z,14Z,17Z)/0:0)], *m/z* 542 [PS(20:5(5Z,8Z,11Z,14Z,17Z)/0:0)] and *m/z* 544 [PS(20:4(5Z,8Z,11Z,14Z)/0:0)], was statistically significant in the semi-quantitative analysis when compared to the control uninfected culture, as shown in Fig 3. PS seems to play a crucial role during virus infection [8, 11, 34]. The viral infection, in turn, induces asymmetry in plasma membrane composition, similar to the abovementioned apoptotic process, facilitating phagocyte recognition of infected cells [37–40]. In addition, viral envelope phosphatidylserine are essential for viral replication, mediating biding to target cells or phagocytosis of viral particles, such as demonstrated for West Nile, Dengue and Ebola viruses [8, 11]. Several recognition mechanisms of apoptotic cells by phagocytes were described during the infection of several viruses, such as those that recognize CD300a, TIM and TAM receptors, observed in DENV infection [8, 9]. In addition, the availability of PS in the outer side of circulating infected-cells signals through TAM receptors to tighten cell junctions, modulating blood-brain barrier integrity and preventing virus transit across brain microvascular endothelial cells during viral infection [41].

### Phosphatidylethanolamines (PE)

PE, as well as PS, is an anionic lipid that is normally found in the inner side of plasma membranes [33, 34], and are synthesized via the decarboxylation reaction of PS [42, 43]. It is the second most abundant lipid class in plasma membranes, after phosphatidylcholines (PC) [15]. PE is externalized upon any cellular biochemical imbalance [33, 34], probably by the same mechanism of PS, indicating that apoptosis is underway [44]. As for PS, the three PE elected as markers for the control group (*m/z* 757 [PE(18:2(9Z,12Z)/19:0)], *m/z* 773 [PE(20:0/18:1(9Z))] and *m/z* 779 [PE(16:0/22:6(54Z,7Z,10Z,12E,16Z,19Z)(14OH))]) did not present significant differences in content when compared to the ZIKV-infected group (data not shown). Since PS and PE play the same role during apoptosis signaling [44], the same premises presented above may explain the lack of significance in this comparison.

PE, as well as PS, is a substrate for TAM [13] and CD300a [8] receptors, involved in viral internalization into the cell. The species elected as marker for the ZIKV-infected group, *m/z* 632 [PE(12:0/16:1(9Z))], presented statistically significant differences in the semi-quantitative analysis (Fig 3), probably due to a cellular response mechanism that uses this particular PE as an alert signal for phagocytes upon viral infection [44]. Another potential explanation is that this PE is a component of the viral envelope during its replication (Fig 4).

### Phosphatidylcholines (PC)

PC is the most abundant plasma membrane phospholipid, and its distribution, as opposed to PE and PS, is on the outer side of the membrane [45]. PC was only elected as marker for the ZIKV-infected group, which presented greater amount of this compound when compared to the -uninfected one (Table 1). This is in agreement with similar findings reported for DENV-infected cell cultures [14, 15]. The 4 PC elected as markers for ZIKV infection (*m/z* 530 [PC(19:3(10Z,13Z,16Z)/0:0)], *m/z* 536 [PC(19:0/0:0)], *m/z* 556 [PC(6:2(3E,5E)/14:2(11E,13E))] and *m/z 558* [PC(6:2(3E,5E)/14:1(13E))]) also presented statistically significant quantitative differences (Fig 3), evidencing that there is a greater amount of these lipids in the ZIKV-infected group, compared to the control one. Two out of the PC elected as markers of ZIKV have already been previously associated to cell response against infectious agents. The ion at *m/z* 530, which corresponds to PC(19:3(10Z,13Z,16Z)/0:0) has already been found on the metabolome of bile acids in patients with altered microbiota and pyloric sphincter [46]. Ion *m/z* 536, corresponding to PC(19:0/0:0) was reported in a lipidomics study that analyzed inflammatory mediators as potential biomarkers for bacteremia [47]; however, in this study made with blood plasma, this species was implicated as a marker of uninfected, bacteremia-free patients, as opposed to what was found in our *in vitro* study. Such difference may be due to either the pathogen used in the study or the study type (*in vivo* vs. *in vitro*); in either case, both lipids are associated with a cell response to an infectious agent, whose mechanism is yet to be elucidated [46, 47].

Furthermore, another study has demonstrated that infections caused by positive RNA viruses, such as ZIKV, promote an increase or accumulation of PC—which is utilized in the formation of viral replication complex (VRC), and also for the replication of the virus in the VRC [14]–a potential explanation for why those are the markers displaying the greatest amounts in ZIKV-infected cells when compared to the -uninfected ones (1.74-fold for ion 530 *m/z*, 1.96-fold for ion 536 *m/z*, 2.15-fold for ion 556 *m/z* and 1.94-fold for ion 558 *m/z*).

### Other lipid classes

Three other lipids were identified as markers: two for the control group, a phosphatidic acid at *m/z* 409, PA(16:0/0:0), and a triacylglycerol at *m/z* 925, TG(18:2(9Z,12Z)/20:4(5Z,8Z,11Z,14Z)/20:4(5Z,8Z,11Z,14Z)); and one for the ZIKV group, a diacylglycerol at *m/z* 925, DG(16:1(9Z)/16:1(9Z)/0:0). The latter may be explained due to the increased metabolic activity of ZIKV-infected cells, since DG is part of PE and PC synthesis, as well as Kennedy Pathways [28, 48]. Once again, the other two elected markers for the control group did not present significant differences in the semiquantification when compared to the ZIKV group, indicating that these species may be common in both cell cultures.

Our findings suggest that PS, PE, SL and PC specific-lipids are putative biomarkers of ZIKV infection in C6/36 mosquito cells. These results are in line with previous lipidomics studies of virus-infected cells. Previous contributions, especially those that studied the same cell line (C36/6 mosquito cells) infected with DENV, have observed increased profiles of lipids such as phosphaditylcholines (PC), phosphatidylethanolamines (PE) and phosphatidylserines (PS) compared to the control cells [15], which are exactly the same view in our study. Furthermore, other recent study also demonstrated higher level of lipid classes of PC, PE, PS and phosphatidylglycerols (PG) during infection of brome mosaic virus (BMV) in YPH500 yeast [14], indicating that these lipids are intimately involved with viral infection/replication in different cell types and organisms. Hence, our results provide valuable information on metabolites that may be used as therapeutic targets for further studies.

## Supporting Information

S1 Fig PLS-DA model validation by permutation tests based on prediction accuracy. The p value based on permutation is p<5e-04 (0/2000).
(DOC)

S2 Fig PLS-DA model validation by permutation tests based on prediction accuracy during training. The p value based on permutation is p < 5e-04 (0/2000).
(DOCX)

S3 Fig Semiquantitative analysis of characteristic lipids in control cells. The bars represent a confidence interval of 95%. Note that there is no statistically significant difference between groups.
(DOCX)
