# Supplementary material for: A Lipidomics Approach in the Characterization of Zika-Infected Mosquito Cells: Potential Targets for Breaking the Transmission Cycle
Source: PLoS One. 2016 Oct 10;11(10):e0164377. doi: 10.1371/journal.pone.0164377 (PMC5056752; doi:10.1371/journal.pone.0164377)
Supplement: S2 Fig — The p value based on permutation is p < 5e-04 (0/2000). (DOCX) [file pone.0164377.s002.docx]

**
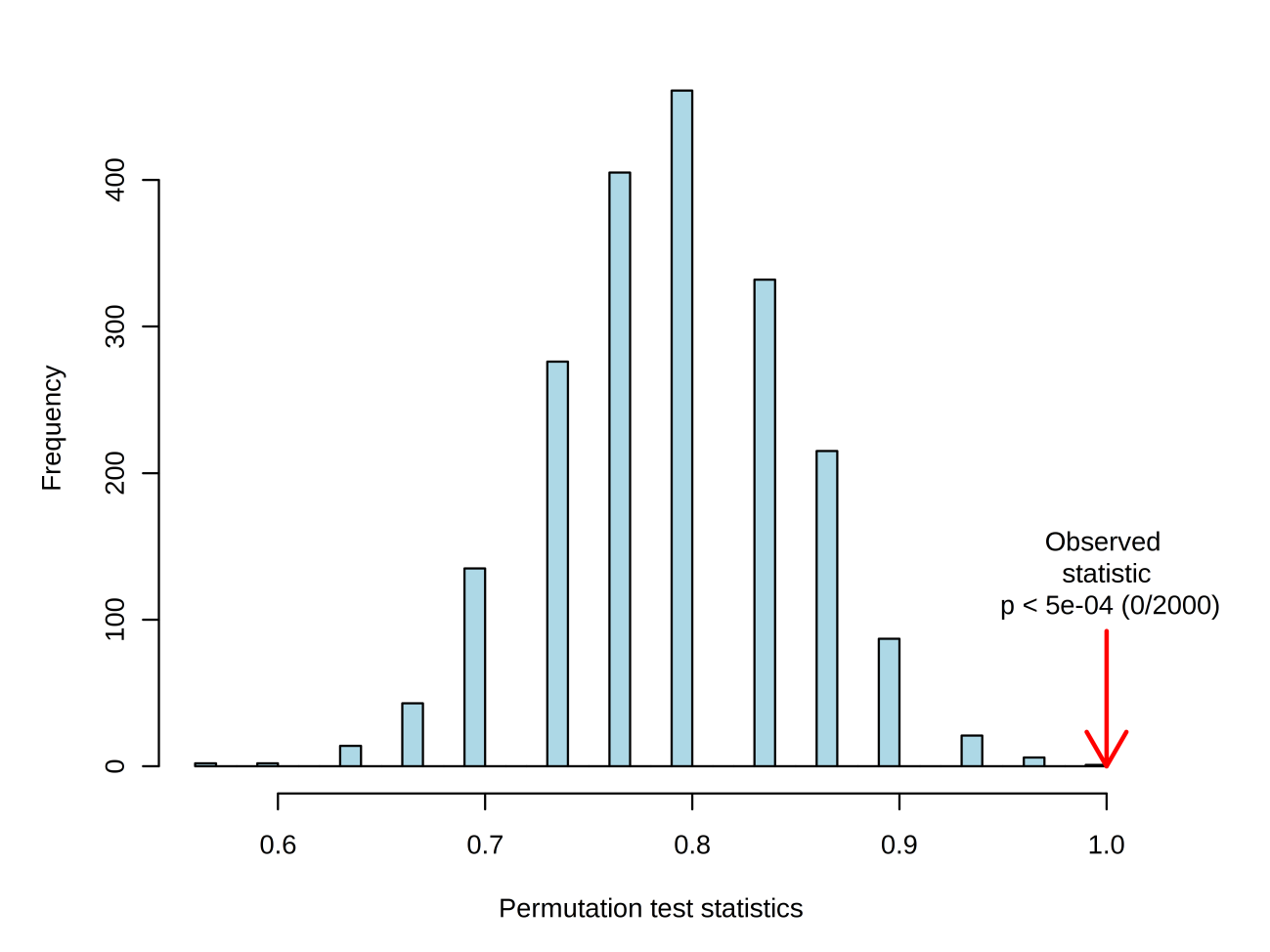
**

**S2 Fig:** PLS-DA model validation by permutation tests based on prediction accuracy during training. The p value based on permutation is p < 5e-04 (0/2000).
