# Supplementary material for: A Lipidomics Approach in the Characterization of Zika-Infected Mosquito Cells: Potential Targets for Breaking the Transmission Cycle
Source: PLoS One. 2016 Oct 10;11(10):e0164377. doi: 10.1371/journal.pone.0164377 (PMC5056752; doi:10.1371/journal.pone.0164377)
Supplement: S3 Fig — The bars represent a confidence interval of 95%. Note that there is no statistically significant difference between groups. (DOCX) [file pone.0164377.s003.docx]

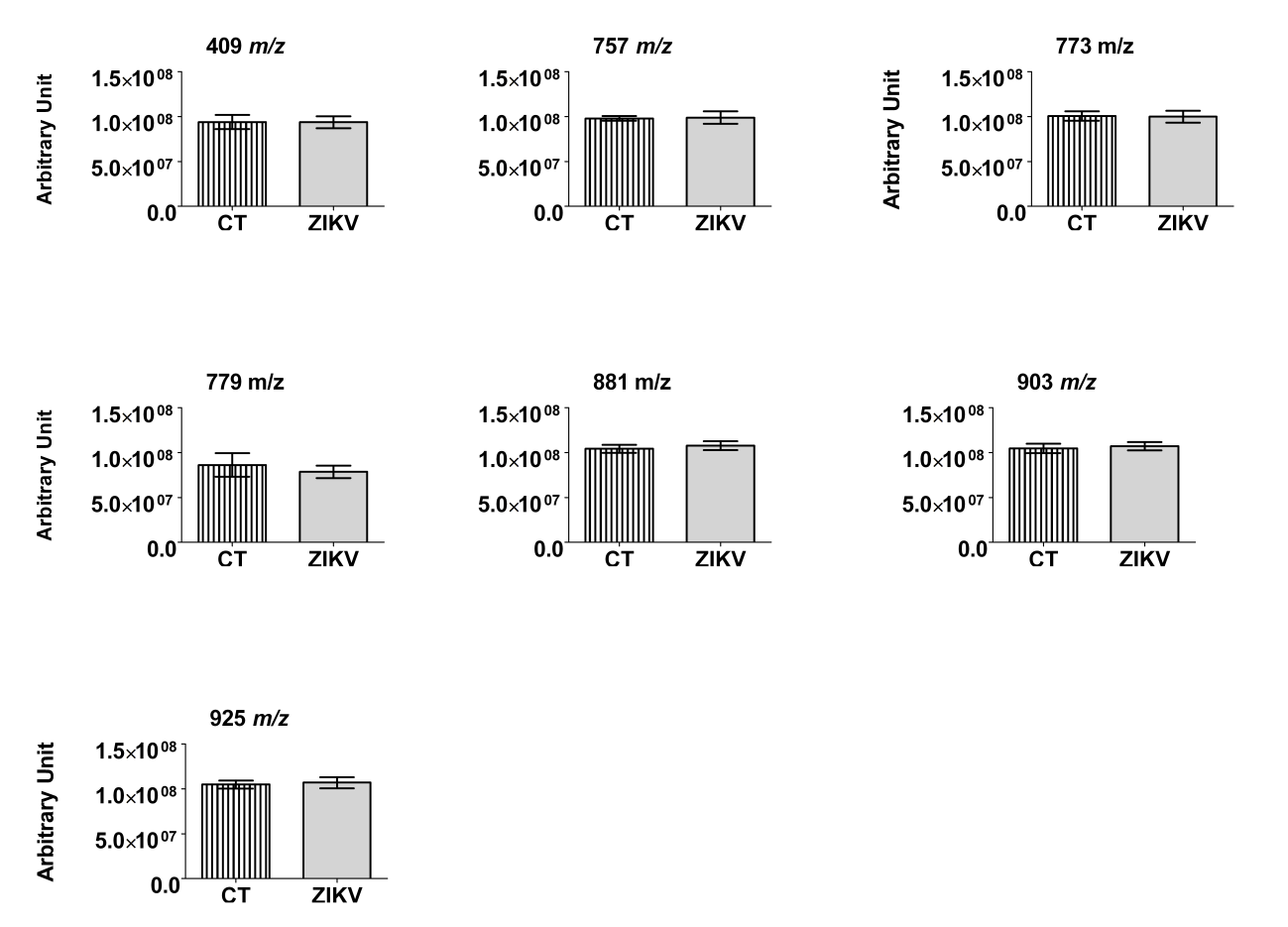


**S3 Fig:** Semiquantitative analysis of characteristic lipids in control cells. The bars represent a confidence interval of 95%. Note that there is no statistically significant difference between groups.
